# Risk Factors of Transient Neurological Deficits and Perioperative Stroke after Revascularization in Patients with Moyamoya Disease

**DOI:** 10.3390/brainsci12101285

**Published:** 2022-09-23

**Authors:** Xincheng Zhang, Yiping Yang, Chao Gan, Xuejun He, Yanchao Liu, Yimin Huang, Xiaopeng Ma, Sheng Wang, Kai Shu, Ting Lei, Huaqiu Zhang

**Affiliations:** Department of Neurosurgery, Tongji Hospital, Tongji Medical College, Huazhong University of Science and Technology, Wuhan 430030, China

**Keywords:** moyamoya disease, revascularization, complications, transient neurological deficits

## Abstract

Objective: To analyze the risk factors of transient neurological deficits (TND) and perioperative stroke in patients with MMD after extracranial–intracranial revascularization. Methods: A retrospective analysis of the clinical data of 183 patients with MMD undergoing 203 EC–IC bypass operation procedures from January 2018 to August 2020. According to whether TND and stroke occurred within 14 days after operation, univariate analysis and multivariate logistic regression were used. Results: TND occurred in 26 cases (12.8%) of revascularization. The results of the univariate analysis showed that history of diabetes, multiple episodes of preoperative symptoms, lesions involving the posterior circulation, and high postoperative blood pressure are the risk factors of TND. Further multivariate logistic regression analysis showed that multiple episodes of preoperative symptoms (*p* = 0.016) and lesions involving the posterior circulation (*p* = 0.014) are the independent risk factors for TND. Perioperative stroke occurred in 12 cases (5.9%). The results of the univariate analysis showed that older age, history of hypertension, preoperative cerebral infarction as the main symptom, lesions involving the posterior circulation, and high perioperative blood pressure are the risk factors of perioperative stroke. The results of multivariate logistic regression analysis showed that preoperative cerebral infarction as the main symptom (*p* = 0.015) is an independent risk factor for perioperative stroke. The occurrence of perioperative complications was not related to the improvement of follow-up mRS (Modified Rankin Scale) score and long-term cerebral rehemorrhage. Conclusions: Clinically, patients with MMD have multiple episodes of preoperative symptoms, lesions involving the posterior circulation, and preoperative cerebral infarction and should be attached when undergoing revascularization.

## 1. Introduction

Moyamoya disease is a chronic progressive cerebral vascular occlusive disease of unknown causes [1]. With the improvement of diagnostic technology, the reported incidence of moyamoya disease is increasing, especially in countries in East Asia [2,3]. Due to its amazing protective effect, EC–IC bypass has become a first-line treatment for patients with MMD [4]. Surgery methods mainly include direct, indirect, and combined revascularization. Combined revascularization can not only increase intracranial blood flow immediately but can also form effective collateral circulation in the long term, which has been widely performed all over the world [5,6]. However, as a kind of preventive surgery, complications such as new current ischemic and hemorrhagic strokes, subdural effusion, poor scalp healing, and transient neurological deficits (TND) are still major problems for neurosurgeons [7,8]. In this study, we studied the two most common complications, TND and perioperative stroke, to analyze the underlying mechanism and provide a reference for clinical work in the future.

## 2. Methods 

### 2.1. Patient Selection

A total of 183 patients and 203 cases of MMD revascularization were included. Inclusion criteria: ① Patients with MMD admitted to our hospital from January 2018 to August 2020. ② Patients with MMD who meet the diagnostic criteria released by the Research Committee on the Pathology and Treatment of Spontaneous Occlusion of the Circle of Willis from the Ministry of Health and Welfare of Japan in 2012 [5]. ③ Patients who underwent revascularization. Exclusion criteria: ① Patients with autoimmune diseases such as systemic lupus erythematosus, antiphospholipid antibody syndrome, Sjogren’s syndrome, hyperthyroidism, tuberous sclerosis, Marfan syndrome, and so on. ② Patients not diagnosed by DSA or MRA. ③ Patients were followed up less than 3 months after discharge, including death.

### 2.2. Clinical Data

The data of 21 clinical indicators were collected, including gender, age, history of hypertension, diabetes and aneurysm, the main clinical symptom of patients, preoperative attack times, interval between the last attack and operation, stage classification of MMD, lesions involving the posterior circulation, admission mRS score, operation method, operation side, times of bypass, duration of surgery, cerebrovascular occlusion times, intraoperative blood loss, intraoperative PCO_2_, perioperative blood pressure (preoperative, intraoperative, and postoperative), 24 h cranial drainage volume after operation, perioperative complications after operation, follow-up mRS score, and long-term cerebral rehemorrhage (Table 1 and Table 2).

### 2.3. Definition of Complications

Transient neurological deficits (TND): New symptoms of local neurological dysfunction during the perioperative period after operation, including aphasia, limb weakness, sensory abnormality, epilepsy, vomiting, vertigo, facial paralysis, blurred vision, drinking water cough, and disturbance of consciousness. There was no new responsible focus of stroke in an imaging examination, and the symptoms recovered within 14 days or 1 month at the latest. Perioperative stroke: New ischemic and hemorrhagic stroke events within 14 days after operation must have new responsible lesions defined upon imaging examination. There is no intersection between perioperative stroke and TND. Patients with TND first and then stroke were recorded as within the perioperative stroke group because it was impossible to distinguish TND from the precursor of stroke.

Long-term cerebral hemorrhage: New hemorrhagic stroke incident one month after surgery, which must have imaging (CT) evidence and excludes cerebral hemorrhages caused by trauma, ruptured aneurysm, and other causes that are not related to MMD.

### 2.4. Definition of Influencing Factors

Main clinical symptom of patients: Refers to the most obvious symptom caused by MMD. Patients with both a preoperative history of cerebral infarction and cerebral hemorrhage were included in the cerebral hemorrhage group because cerebral hemorrhage is often more serious. 

The interval between the last attack and operation: Patients in the acute operation group underwent operation within 1 month after the onset of cerebral infarction or within 3 months after the onset of a cerebral hemorrhage. Patients in the stable operation group were operated on within 1 to 6 months after the onset of cerebral infarction, or within 3 to 6 months after the symptoms of a cerebral hemorrhage. Patients who underwent operation more than 6 months after the onset of stroke symptoms were regarded as members of the long-term operation group.

MMD stage: Refers to the staging on the side of the operation. Suzuki stage 1–2 or MRA score 0–1 is in the early stage, Suzuki stage 3–4 or MRA score 2–7 is in the middle stage, and Suzuki stage 5–6 or MRA score 8–10 is in the late stage [5].

Preoperative blood pressure: Patients previously diagnosed with hypertension did not regularly use antihypertensive drugs, and patients newly diagnosed with hypertension (In the case of no antihypertensive drugs, systolic blood pressure ≥140 mmHg and diastolic blood pressure ≥90 mmHg were measured 3 times in the clinic, not in the same day) were recorded as preoperative hypertension.

Intraoperative blood pressure: Intraoperative high blood pressure was recorded when arterial systolic blood pressure ≥150 mmHg or arterial diastolic blood pressure ≥100 mmHg and the duration was more than 5 min.

Postoperative blood pressure: Systolic blood pressure ≥140 mmHg or diastolic blood pressure ≥90 mmHg measured 3 times, not in the same day, during the perioperative period after operation was regarded as postoperative high blood pressure. PCO_2_ during operation: PCO_2_ < 35 mmHg measured at one-third of the time or recorded twice randomly during the operation was regarded as low PCO_2_ during the operation. PCO_2_ > 45 mmHg measured at one-third of the time or recorded twice randomly during the operation was regarded as high PCO_2_ during the operation.

Cerebrovascular occlusion times: During direct bypass surgery, the time from arterial clamp clamping to completion of anastomosis, removal of arterial clamp, and recanalization of recipient vessels. They were divided into three groups: ≤20 min, 20 min~40 min, and >40 min. Simple indirect bypass surgery was placed into the ≤20 min group because the recipient vessels were not clipped.

### 2.5. Operation

The surgical methods include combined revascularization and indirect revascularization. Indirect revascularization mainly refers to encepho-duro-myo-synangiosis (EDMS), Combined revascularization refers to the simultaneous implementation of extracranial–intracranial bypass surgery + EDMS. Extracranial–intracranial bypass surgery, that is, direct bypass surgery, used a single bypass (using the frontal branch of the superficial temporal artery by default) as the donor vessel, and the M4 segment of the middle cerebral artery as the recipient vessel for revascularization.

### 2.6. Follow-Up Method

Three months after surgery, the patients were followed up. All-inclusive patients were followed up for ≥3 months (MD = 15 months, IQR = 9 months). The follow-up content includes Modified Rankin Scale (mRS) score and whether there is a long-term cerebral hemorrhage incident.

### 2.7. Statistical Method

Statistical analysis of data was performed using IBM SPSS Statistics version 25.0 (IBM Software Group, Chicago, IL, USA). The measurement data use the *K-S test* for normal testing, and the measurement data that do not meet the normal distribution use the *P-P grammar* to determine whether they approximately obey the normal distribution or not. The measurement data which conformed to or approximately conformed to the normal distribution were expressed by mean ± standard deviation ($\bar{x}\pm s$), and the differences between groups were compared by a two independent samples *t-test*. The measurement data that did not conform to normal or approximately normal distributions were expressed by median and Inter-Quartile Range (Md, IQR), and the differences between groups were compared by a *rank-sum test*. The counting data were expressed by the number of cases and the rate. The *chi-square test* or *Fisher’s exact test* was used to compare the differences between groups, and the *Bonferroni correction* was used for pairwise comparison within the group. A *Rank sum test* was used for ordinal variable data. Multivariate analysis was conducted using *binary logistic regression analysis*. *p*-values < 0.05 were considered statistically significant.

## 3. Results

### 3.1. Incidence of Complications

**Figure 1:**
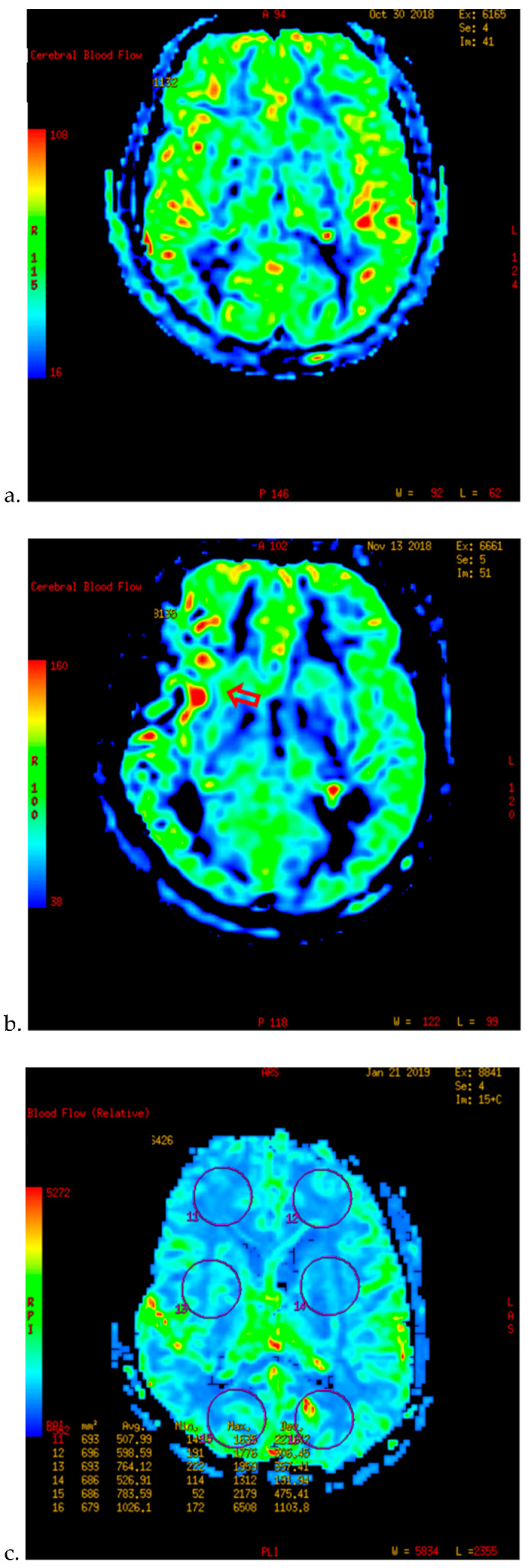
PWI of patients with TND in the perioperative period after operation (example 1). (**a**) The PWI image on the day before operation showed that the cerebral blood flow (CBF) of both sides was basically the same. (**b**) On the 7th day after right combined revascularization, the patient developed epileptic symptoms. PWI examination showed that the CBF in the right operating area was significantly higher than that in the contralateral side on the 13th day after operation. (**c**) The patient’s TND symptoms recovered after more than ten days of repeated attacks. Two months after discharge, PWI showed that the cerebral blood flow of the two hemispheres tended to balance.

**Figure 2:**
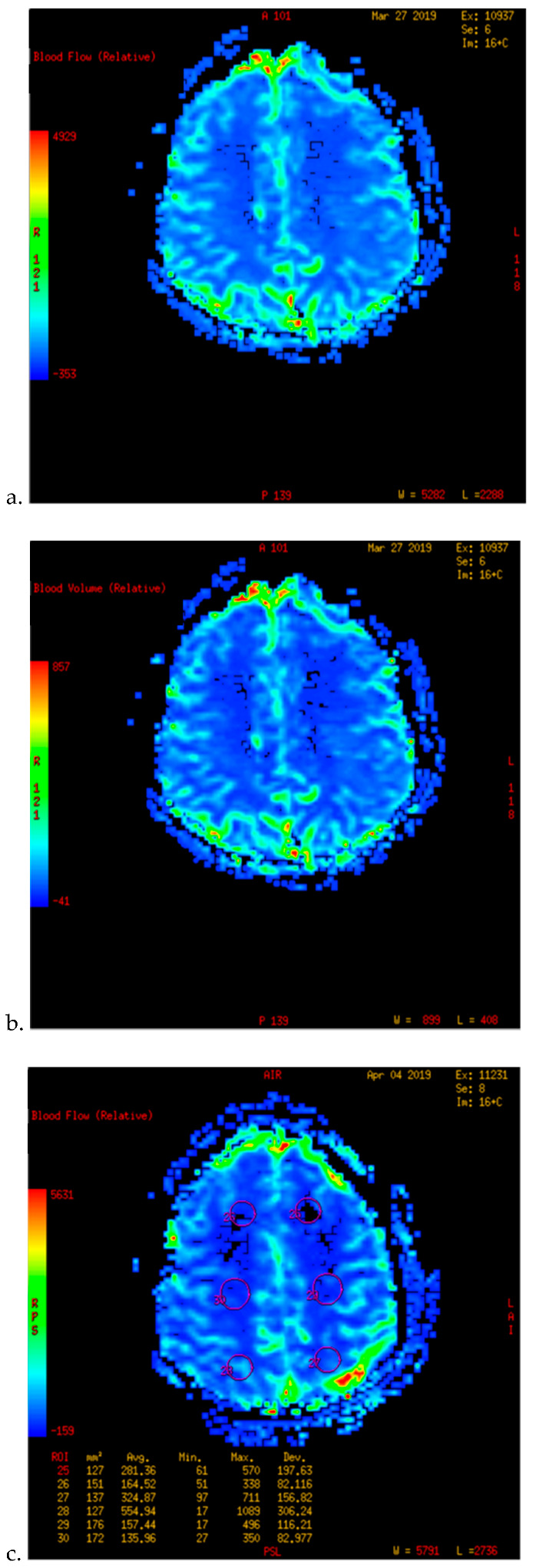

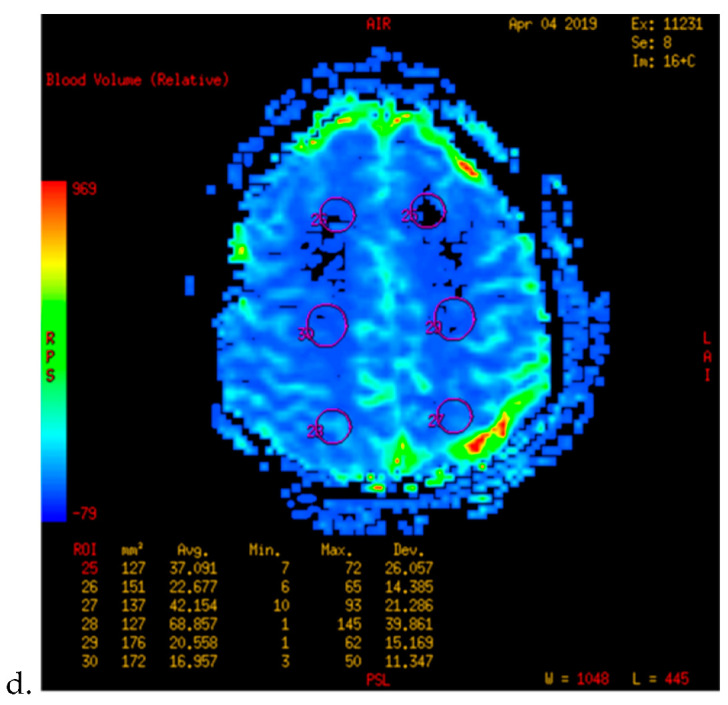
PWI of patients with TND in the perioperative period after operation (example 2). (**a**,**b**) The PWI images of the patients 5 days before operation showed that the cerebral blood flow (CBF) and cerebral blood volume (CBV) of the bilateral hemispheres were basically the same. (**c**,**d**) 3 days after the left combined operation, the patients showed blurred vision and dizziness. 6 days after operation, the PWI images showed that the CBF and CBV of the left parietal occipital lobe were significantly higher than those of the opposite side.

TND occurred in 26 cases (12.8%) after cerebrovascular revascularization, of which aphasia (26.9%) was the most common symptom. The median time of occurrence of TND was 3 days, and the median duration of TND was 6 days. There were 12 cases of perioperative stroke (5.9%) and 8 cases of long-term cerebral hemorrhage (3.9%) (Table 3, Figure 1 and Figure 2).

### 3.2. Statistical Outcomes

Analysis of factors influencing the occurrence of perioperative complications: Univariate analysis showed that history of diabetes, multiple episodes of preoperative symptoms, lesions involving the posterior circulation, and high postoperative blood pressure were the risk factors of postoperative TND (*p* < 0.05). Meanwhile, older age, history of hypertension, the main clinical symptom of patients, lesions involving the posterior circulation, and high perioperative blood pressure are the risk factors of the perioperative stroke (*p* < 0.05). Further multivariate logistic regression analysis showed that multiple episodes of preoperative symptoms (OR = 3.134, CI: 1.236~7.943, *p* = 0.016) and lesions involving the posterior circulation (OR = 0.327, CI: 0.133~0.8, *p* = 0.014) are independent risk factors for TND. Meanwhile, the main clinical symptom of patients was an independent risk factor for perioperative stroke (OR = 0.209, CI: 0.059~0.738, *p* = 0.015), and the pairwise comparison within the main clinical symptom group showed that the incidence of perioperative stroke in the preoperative cerebral infarction group was higher than that in the preoperative cerebral hemorrhage group (*p* < 0.0125) (Table 4 and Table 5).

### 3.3. Follow-Up Results

There was no statistical difference in mRS between the perioperative complication group and the non-complication group (*p* = 0.094). None of the eight patients with long-term cerebral hemorrhage had perioperative complications, so perioperative complications had nothing to do with long-term cerebral hemorrhage (*p* = 0.37) (Table 6).

## 4. Discussion

MMD is a rare cerebrovascular disease characterized by chronic progressive stenosis or occlusion of bilateral internal carotid arteries and secondary to the formation of an abnormal vascular network in the skull base [1]. Its incidence is relatively high in East Asia, and the age distribution of patients conforms to the bimodal distribution; that is, children and young adults have a higher incidence, while females have a higher incidence than men [9,10]. However, the clinical data of our center do not show obvious age bimodal distribution characteristics and gender differences, which is consistent with the reports of some Chinese Studies [11,12], which may be affected by the sample size and the misdiagnoses of moyamoya disease in children. As a first-line therapy of MMD, cerebral revascularization can effectively protect patients [4], but its postoperative complications are more frequent and complex than other diseases undergoing EC–IC bypass surgery [7]. Neurologic deterioration is a common complication of cerebral revascularization for the perioperative period in MMD, which can be divided into TND and stroke according to its reversibility and imaging findings.

### 4.1. The Mechanism of TND

At present, reports on the mechanism and influencing factors of TND are still not comprehensive. It was previously reported that the incidence of TND after cerebral revascularization was about 9.4–38.2% [13,14,15,16,17], compared with 12.8% in this cohort, which is basically consistent with the reported studies. Early research by Iwama et al. [18] reported symptoms similar to TND but classified them as a kind of reversible ischemic neurologic deficit (RIND). They suggested that the mechanism of this symptom was caused by intracranial ischemia and was a precursor of cerebral infarction, but there was no imaging-responsible focus. In 1998, Uno et al. [19] reported for the first time the phenomenon of TND with hyperperfusion on SPECT in patients with MMD after EC–IC bypass, considering that the symptoms of TND in patients with MMD may be related to hyperperfusion. Subsequent scholars found hyperperfusion and local cerebral edema through SPECT and MRI and their time relationship with TND, confirming that EC–IC bypass has the phenomenon of hyperperfusion, which may lead to hyperperfusion syndrome rather than ischemic injury, and this kind of symptom is called symptomatic hyperperfusion [14,15,16]. Later, Hayashi et al. [20] observed a decrease in regional cerebral perfusion far away from the anastomotic stoma on the SPECT of 15 children with TND, suggesting that watershed shift may also be the mechanism leading to TND. The study of Mukerji et al. [21] also proposed that the impairment of the self-regulating function of cerebral blood flow and local hypoperfusion caused by competitive blood flow may also be the cause of TND. The above studies have shown that changes in hemodynamics after EC–IC bypass are a mechanism that occurs in TND, but for indirect bypass, the 2 weeks after surgery can form a significant lateral recirculation, causing hemodynamics to change. However, in our study, TND occurred in two of the seven patients with indirect bypass on the 2nd and 6th day after operation, respectively. The explanation given by Phi et al. [22] is that TND after indirect bypass may also be caused by the transient cortical depression, triggered by mechanical stimulation rather than hyperperfusion. Therefore, can terms symptomatic hyperperfusion and cerebral hyperperfusion syndrome (CHS) be used instead of TND? The answer is no. TND is observed from symptomatology, while CHS is observed from imaging. The occurrence of TND does not necessarily have regional cerebral hyperperfusion, and it is observed that the presence of CHS does not automatically show clinical symptoms. The intersection of TND and CHS is symptomatic hyperperfusion, and CHS is more common than symptomatic hyperperfusion [23]. 

### 4.2. The Mechanism of Perioperative Cerebral Infarction

It was previously reported that the incidence of perioperative cerebral infarction events was 3~10.3% [24,25,26,27,28], and our incidence was 4.9%. It is now believed that the mechanism of perioperative ischemic stroke is related to watershed shift, perioperative management, and even the space-occupying effect of the temporal muscle [29]. Regarding the location of the stroke, surgical stroke is more common; contralateral or bilateral stroke is relatively rare [30]. In our study, 10 patients with perioperative stroke had surgical strokes and 2 patients had bilateral strokes.

### 4.3. The Mechanism of Perioperative Cerebral Hemorrhage

In the past, the incidence of perioperative cerebral hemorrhage was reported as 0.13–3.6% [26,31,32,33], which was similar to our result (0.98%). The occurrence of hemorrhagic stroke is mainly related to hyperperfusion syndrome [33]. In addition, severe transfer of watershed shift can also lead to hemorrhagic stroke. Ischemia–reperfusion injury after EC–IC bypass will increase the risk of bleeding [34]. During the reperfusion of ischemic brain tissue, a variety of substances that can increase vascular permeability, including reactive oxygen species, vascular endothelial growth factor, and protease, may be released, which may lead to an increase in vascular permeability, leakage of plasma and red blood cells, and even vascular rupture and bleeding [35]. 

In this study, we explored the effects of 19 factors on perioperative complications after revascularization of MMD and the relationship between perioperative complications and long-term prognosis. These factors were divided into four types: baseline data of patients, information of MMD, operation and anesthesia management, and perioperative management data.

### 4.4. Baseline Data of Patients

Age: Our study found that older patients had a higher incidence of postoperative stroke events (*p* < 0.05), which may be due to the loss of self-regulating function of cerebral blood flow, or they are more sensitive to cerebral ischemia [12].

History of hypertension: History of hypertension is also related to postoperative stroke events. The accelerated hardening of intracranial vessels and the decrease in cerebrovascular reserve capacity (CVR) in patients with a history of hypertension, coupled with the impact and cutting effect of high-pressure blood flow on the blood vessel wall, make endothelial cells damaged and prone to form microthrombi, which make hypertensive patients more likely to have postoperative stroke events [36].

History of diabetes: We found that history of diabetes is related to TND (*p* < 0.05). Excessive blood glucose interferes with the endogenous cardiovascular nitric oxide system, resulting in a decrease in nitric oxide-dependent endothelial dilation function and a decrease in brain CVR. In addition, hyperglycemia reduces the oxygen delivery capacity of hemoglobin, aggravates brain ischemia, and leads to lactate accumulation and intracellular acidosis, which in turn aggravates cerebral ischemia [37,38].

### 4.5. Moyamoya Disease Information

Main clinical symptom: Previous studies have shown that postoperative CHS is independently associated with preoperative ischemic manifestations [26]. However, our study did not find the relationship between preoperative main clinical symptom and TND, but we found a relationship between preoperative main clinical symptom and postoperative stroke. The incidence of postoperative stroke events in patients with cerebral infarction as the main clinical symptom before operation was higher than that of patients with cerebral hemorrhage as the main symptom before operation (*p* < 0.0125), and 10 cases of postoperative cerebral infarction occurred in patients whose preoperative main clinical symptom was cerebral infarction. However, one of the two patients with postoperative cerebral hemorrhage had cerebral infarction as the main symptom before operation, which indicated that patients with ischemic stroke before operation were more likely to have cerebral infarction after operation. This is consistent with the research of Park et al. [28]. 

Preoperative attack times: Multiple episodes of preoperative symptoms is a relatively clear risk factor, which has been reported by several studies [13,28,39]. Multiple episodes of preoperative symptoms indicate that the compensatory ability of intracranial vessels is obviously impaired and CVR is seriously insufficient, so symptoms appear repeatedly before operation, and it is more difficult to adapt to the sudden increase in blood flow after operation. 

Stage classification of MMD: Zhao et al. [26] believe that the later the Suzuki staging of MMD, the more likely it is to have complications. We believe that some MMD patients in the later stage have formed perfect compensatory collateral vessels such as anterior choroid vessels, posterior circulation, and extracranial vessels [27]; on the contrary, the degree of intracranial ischemia will be alleviated because of the powerful compensation system. Similarly, our results do not show the relationship between stage classification of MMD and complications. 

Lesions involving the posterior circulation: In this study, the lesions involving the posterior circulation were considered to be related not only to TND but also to postoperative stroke. Posterior circulation is one of the most important intracranial compensatory vessels in patients with MMD. Typical MMD first involves the internal carotid artery system; the posterior communicating artery will open, and the vertebrobasilar artery can supply blood to the anterior circulation through the cerebral arterial circle. According to the Suzuki staging system, at stage 4, the cerebral arterial circle or even the posterior cerebral artery are occluded, and the vertebrobasilar artery system can only play a compensatory role through the new collateral circulation. At stage 6, the blood supply of the brain completely depends on the collateral circulation formed by the external carotid artery and the vertebrobasilar artery system [5,40]. However, in fact, not all MMD patients can successfully form sufficient posterior circulation in lateral compensatory vessels. Bao et al. [12] have observed that about 30% of MMD patients have posterior circulation stenosis or even occlusion. Once the posterior circulation is occluded, the narrow internal carotid artery system will lose an important source of compensatory blood vessels. In the face of sudden high-flow superficial temporal artery (STA) blood flow, the already fragile intracranial blood structure may be disturbed by the new bypass vessels, and it is not difficult to understand that the incidence of postoperative complications will increase [28,41]. 

### 4.6. Surgery and Anesthesia Management

Operation method: In theory, the direct bypass has a faster impact on intracranial blood flow, so its perioperative complications should be higher in number than that of indirect bypass, but a meta-analysis shows that there is no significant difference in direct and indirect bypass complications [42]. The center will only use indirect bypass surgery for those whose basic physical conditions are too poor to tolerate the operation and those who cannot find a suitable recipient vessel, so our results show that the incidence of complications of the indirect bypass is slightly higher, but the difference is not statistically significant. 

PCO_2_: Under physiological conditions, intracranial vessels dilate in hypercapnia and contract in hypocapnia, but for patients with MMD, the intracranial diseased vessels have already dilated to the maximum extent, so an increase in PCO_2_ will only dilate the vessels without lesions, resulting in the phenomenon of blood theft [43]. This effect of PCO2 is usually seen in perioperative cerebral infarction caused by crying and hyperventilation in children with MMD [43]. 

Other factors, such as intraoperative blood loss, urine volume, and hemoglobin concentration, represent the effective circulating blood volume, which is mainly involved in the occurrence of ischemic stroke. In addition, Hwang et al. [44] also found that an increase in postoperative white blood cell count in blood was related to TND. However, the significance of these factors was not found in this study.

### 4.7. Perioperative Management Data

Blood pressure: Our study found that preoperative, intraoperative, and postoperative high blood pressure were associated with the occurrence of postoperative stroke, and postoperative high blood pressure was also associated with TND. An increase in blood pressure during and after operation will increase the perfusion pressure of donor and recipient blood vessels, and make the contradiction between the two kinds of blood flow more intense, which will not only aggravate hyperperfusion injuries, but also shift the watershed, and even trigger cerebral infarction in the contralateral hemisphere [30]. Fujimura et al. [36] reported that controlling systolic blood pressure at 110 mmHg~130 mmHg can reduce the risk of symptomatic hyperperfusion from 24.7% to 6.7%. However, blindly reducing blood pressure can increase the risk of cerebral infarction. Therefore, in the future, large sample studies are still needed to guide the perioperative blood pressure management of MMD patients after surgery [36,45,46]. 

The multivariate logistic regression analysis showed that multiple episodes of preoperative symptoms (*p* = 0.016) and lesions involving the posterior circulation (*p* = 0.014) are the independent risk factors for TND, and preoperative cerebral infarction as the main clinical symptom of MMD (*p* = 0.015) is an independent risk factor for perioperative stroke in patients with MMD. When this kind of high-risk patient is encountered clinically, indocyanine green video angiography can be used to select appropriate recipient vessels during operation to reduce the occurrence of postoperative hyperperfusion syndrome [47,48,49]. Additionally, we believe that intensive blood pressure-lowering therapy should not be blindly carried out after the occurrence of TND, because for TND with watershed shift, blindly lowering blood pressure may lead to ischemic stroke at the watershed [50]. On the contrary, for patients with new cerebral infarction caused by a watershed shift, blindly expanding blood volume and increased blood pressure may induce the emergence of a high-perfusion area. It is suggested that the postoperative cerebral blood flow of high-risk patients should be measured and the treatment plan should be decided after comprehensive consideration [43]. Moreover, after cerebral revascularization in patients with MMD, a large amount of free radical will be produced due to ischemia–reperfusion injury, which will aggravate damage caused by hyperperfusion. Edaravone [51], a free radical scavenger, and minocycline [52], an inhibitor of matrix metalloproteinase-9 (MMP-9), have been proved to be effective on perfusion disorders after cerebral vascular revascularization.

### 4.8. Limitations

Finally, this study has some limitations: (1) All 183 patients included in this study were from a single center with geographical restrictions. (2) The blood pressure and PCO_2_ levels observed in the study are not the results of continuous monitoring and cannot be well used to represent the whole perioperative period. (3) For a portion of the postoperative stroke events, we cannot clearly judge the time relationship between blood pressure changes and stroke. There may be a situation that reactive blood pressure rises after stroke. We did not collect blood pressure information after complications, as far as possible, to avoid the occurrence of this kind of error. 

## 5. Conclusions

Our study found that multiple episodes of preoperative symptoms and lesions involving the posterior circulation were independent risk factors for postoperative TND, and preoperative cerebral infarction as the main clinical symptom of MMD was an independent risk factor for postoperative stroke. This suggests that more attention should be paid to patients with multiple episodes of preoperative symptoms, cerebral infarction as the main clinical symptom, and DSA angiography showing stenosis or occlusion of the posterior circulation because they are at high risk of postoperative TND and stroke. It is suggested that SPECT (single-photon emission computed tomography), CTP (computed tomography perfusion), and PWI (perfusion-weighted imaging) should be used to predict and evaluate the imbalance of cerebral perfusion in advance. During the follow-up, it was found that there was no correlation between the occurrence of postoperative complications and the improvement of long-term prognosis and long-term cerebral rehemorrhage.

## Figures and Tables

**Table 1:** Baseline Characteristics of 203 cases of revascularization of MMD.

Characteristics	n (%)
Gender	
Male	110 (54.2)
Female	93 (45.8)
Age ($\bar{x}\pm s$, years)	46.76 ± 11.15
History	
Hypertension	72 (35.5)
Diabetes	25 (12.3)
Aneurysm	22 (10.8)
Moyamoya disease information	
Main clinical symptom	
Cerebral hemorrhage	92 (45.3)
Cerebral infarction	54 (26.6)
TIA	31 (15.3)
Chronic cerebral ischemia	26 (12.8)
Preoperative attack times	
One time	160 (78.8)
Two times and more	43 (21.2)
Interval between the last attack and operation	
Acute operation	41 (20.2)
Stable operation	94 (46.3)
Long-term operation	68 (33.5)
Stage classification	
Early stage	16 (7.9)
Middle stage	142 (70)
Late stage	45 (22.2)
Lesions involving the posterior circulation	54 (26.6)
Admission mRS score ($\bar{x}\pm s$, marks)	1.82 ± 1.02
Follow-up mRS score ($\bar{x}\pm s$, marks)	1.34 ± 1.18

**Table 2:** Surgical information of 203 cases of revascularization of MMD.

Information	n (%)
Operation method	
EDMS + STA–MCA bypass	196 (96.6)
EDMS	7 (3.4)
Operation side	
Left	95 (46.8)
Right	108 (53.2)
Time of bypass	
First time	172 (84.7)
Second time	31 (15.3)
Duration of surgery [(Md, IQR), minute]	218 (71)
Intraoperative blood loss ($\bar{x}\pm s$, mL)	138.77 ± 129.76
Intraoperative PCO_2_	
Low	40 (19.7)
Normal	161 (79.3)
High	2 (1)
Perioperative blood pressure	
Preoperative	
Normal	179 (88.2)
High	24 (11.8)
Intraoperative	
Normal	186 (91.6)
High	17 (8.4)
Postoperative	
Normal	161 (79.3)
High	42 (20.7)
24 h cranial drainage volume after operation ($\bar{x}\pm s$, mL)	161.63 ± 81.09

**Table 3:** Summary of 46 cases of Postoperative Complications.

Complications	Total (case)	n (%)	Dominant Hemispheric Surgery (%)
Perioperative period			
TND	26 (12.8%)		
Aphasia		7 (26.9)	7 (100)
Epilepsy		4 (15.4)	1 (25)
Muscle weakness + Aphasia		3 (11.5)	3 (100)
Severe vomiting + Vertigo		3 (11.5)	0 (0)
Muscle weakness		2 (7.7)	0 (0)
Central facial paralysis		2 (7.7)	0 (0)
Consciousness disorders		2 (7.7)	1 (50)
Acroparesthesia		1 (3.8)	0 (0)
Blurred vision		1 (3.8)	1 (100)
Drinking water cough + Epilepsy		1 (3.8)	0 (0)
Stroke	12 (5.9%)		**Surgical side hemisphere (%)**
Infarction		10 (83.3)	7 (70)
Hemorrhage		2 (16.7)	2 (100)
Long-term			
Cerebral rebleeding	8 (3.9%)		2 (25)

**Table 4:** The univariate analysis of perioperative complications after revascularization in 203 operations.

Factors	TND			Perioperative Stroke		
	TND (+) n (%) (n = 26)	TND (-) n (%) (n = 177)	*p* Value	Stroke (+) n (%) (n = 12)	Stroke (−) n (%) (n = 191)	*p* Value
Gender (male/female) ^†^	13/13	97/80	0.646	8/4	102/89	0.371
Age ($\bar{x}\pm s$, years) ^‡^	43.2 ± 12.9	47.3 ± 10.8	0.081	56 ± 8.7	46.2 ± 11.1	0.003 *
History ^†^						
Hypertension	7 (26.9)	65 (36.7)	0.329	8 (66.7)	64 (33.5)	0.044 *
Diabetes	7 (26.9)	18 (10.2)	0.035 *	4 (33.3)	21 (11)	0.067
Aneurysm	2 (7.7)	20 (11.3)	0.83	3 (25)	19 (9.9)	0.251
Main clinical symptom ^†^			0.328			<0.001 *
Cerebral hemorrhage	9 (34.6)	83 (46.9)		1 (8.3)	91 (47.6)	
Cerebral infarction	7 (26.9)	47 (26.6)		11 (91.7)	43 (22.5)	
TIA	7 (26.9)	24 (13.6)		0 (0)	31 (16.2)	
Chronic cerebral ischemia	3 (11.5)	23 (13)		0 (0)	26 (13.6)	
Preoperative attack times ^†^			0.005 *			0.976
One time	15 (57.7)	145 (81.9)		10 (83.3)	150 (78.5)	
Two times and more	11 (42.3)	32 (18.1)		2 (16.7)	41 (21.5)	
Interval between the last attack and operation ^§^			0.876			0.237
Acute operation	5 (19.2)	36 (20.3)		2 (16.7)	39 (20.4)	
Stable operation	12 (46.2)	82 (46.3)		9 (75)	85 (44.5)	
Long-term operation	9 (34.6)	59 (33.3)		1 (8.3)	67 (35.1)	
Stage classification ^§^			0.086			0.064
Early stage	1 (3.8)	15 (8.5)		0 (0)	16 (8.4)	
Middle stage	16 (61.5)	126 (71.2)		7 (58.3)	135 (70.7)	
Late stage	9 (34.6)	36 (20.3)		5 (41.7)	40 (20.9)	
Lesions involving the posterior circulation ^†^	13 (50)	41 (23.2)	0.004 *	7 (58.3)	47 (24.6)	0.026 *
Admission mRS score ^§^			0.136			0.073
0~1	13 (50)	62 (35)		2 (16.7)	73 (38.2)	
2~3	12 (46.2)	103 (58.2)		8 (66.7)	107 (56)	
≥4	1 (3.8)	12 (6.8)		2 (16.7)	11 (5.8)	
Operation method ^II^			0.221			1
EDMS + STA–MCA bypass	24 (92.3)	172 (97.2)		12 (100)	184 (96.3)	
EDMS	2 (7.7)	5 (2.8)		0 (0)	7 (3.7)	
Operation side ^†^			0.726			0.819
Left	13 (50)	82 (46.3)		6 (50)	89 (46.6)	
Right	13 (50)	95 (53.7)		6 (50)	102 (53.4)	
Time of bypass ^†^			0.784			0.27
First time	23 (88.5)	149 (84.2)		12 (100)	160 (83.3)	
Second time	3 (11.5)	28 (15.8)		0 (0)	31 (16.2)	
Duration of surgery [(Md, IQR), minute] ^§^	232 (65)	243 (77)	0.179	232 (101)	217 (64)	0.201
Cerebrovascular occlusion times ^†^			0.927			0.058
<20 min	9 (34.6)	57 (32.2)		3 (25)	72 (37.7)	
20~40 min	14 (53.8)	95 (53.7)		5 (41.7)	99 (51.8)	
>40 min	3 (11.5)	25 (14.1)		4 (33.3)	20 (10.5)	
Intraoperative blood loss ($\bar{x}\pm s$, mL) ^§^	126.9 ± 145.8	140.5 ± 127.6	0.399	162.5 ± 143.2	137.3 ± 129.1	0.406
Intraoperative PCO_2_ ^§^			0.115			0.596
<35 mmHg	8 (30.8)	32 (18.1)		3 (25)	37 (19.4)	
35~45 mmHg	18 (69.2)	143 (80.8)		9 (75)	152 (79.6)	
>45 mmHg	0 (0)	2 (1.1)		0 (0)	2 (1)	
Perioperative blood pressure ^†^						
High preoperative blood pressure	6 (23.1)	18 (10.2)	0.115	5 (41.7)	19 (9.9)	0.005 *
High intraoperative blood pressure	3 (11.5)	14 (7.9)	0.807	4 (33.3)	13 (6.8)	0.007 *
High postoperative blood pressure	11 (42.3)	31 (17.5)	0.004 *	8 (66.7)	34 (17.8)	<0.001 *
24 h cranial drainage volume after operation ($\bar{x}\pm s$, mL) ^‡^	161.9 ± 77.4	161.6 ± 81.8	0.984	165 ± 65.9	161.4 ± 82.1	0.882

^†^ *Chi-square test* was used. ^‡^ *t-test* was used. ^§^ *rank-sum test* was used. ^II^ *Fisher’s exact test* was used. * *p* < 0.05.

**Table 5:** The multivariate analysis of perioperative complications after revascularization in 203 operations.

Risk Factors	*B* Value	OR	95% CI	*p* Value
TND				
History of diabetes	−0.806	0.446	0.142~1.401	0.167
Multiple episodes of preoperative symptoms	1.142	3.134	1.236~7.943	0.016 *
Lesions involving the posterior circulation	−1.119	0.327	0.133~0.8	0.014 *
High postoperative blood pressure	0.831	2.296	0.868~6.077	0.094
Perioperative stroke				
Older age	0.06	1.061	0.988~1.141	0.104
History of hypertension	−0.048	0.953	0.146~6.229	0.96
Main clinical symptom	−1.565	0.209	0.059~0.738	0.015 *
Lesions involving the posterior circulation	−1.353	0.258	0.062~1.079	0.064
Perioperative blood pressure				
High preoperative blood pressure	−0.229	0.795	0.106~5.942	0.823
High intraoperative blood pressure	0.804	2.235	0.351~14.237	0.395
High postoperative blood pressure	1.074	2.928	0.585~14.645	0.191

*B* value: partial regression coefficient. OR: odds ratio. 95%CI: 95% confidence interval of odds ratio. * *p* < 0.05.

**Table 6:** Effect of perioperative complications on long-term prognosis.

	Changes in Long-Term Prognosis	
	Improvement of Prognosis ^¶^	Deterioration of Prognosis ^#^
Perioperative complications		
Occurred	30 (78.9)	8 (21.1)
Did not occur	149 (90.3)	16 (9.7)
*p* value	0.094	

^¶^ Follow-up mRS score—Admission mRS score ≤ 0. ^#^ Follow-up mRS score—Admission mRS score > 0.

## Data Availability

The data presented in this study are available in article.
